# Ibuprofen vs. Acetaminophen After Delivery in Women with Hypertensive Disorders of Pregnancy: An Updated Systematic Review and Meta-Analysis of Randomized Controlled Trials

**DOI:** 10.3390/jcm15114042

**Published:** 2026-05-23

**Authors:** Jelena Cumic, Radomir Anicic, Mladen Mirkovic, Jovana Ristic, Milica Karadzic Kocica, Danka Mostic, Srdjan Masic, Natasa Milic

**Affiliations:** 1 Institute for Cardiovascular Diseases “Dedinje”, 11000 Belgrade, Serbia; jelena.cumic@gmail.com (J.C.); jovanaristic00@gmail.com (J.R.); milica.karadzic@gmail.com (M.K.K.); 2 Faculty of Medicine, University of Belgrade, 11000 Belgrade, Serbia; radomir.anicic@gmail.com (R.A.); mladenm24mirkovic@gmail.com (M.M.); mosticd@gmail.com (D.M.); 3 Clinic for Gynecology and Obstetrics “Narodni Front”, 11000 Belgrade, Serbia; 4 Clinic for Gynecology and Obstetrics, University Clinical Centre of Serbia, 11000 Belgrade, Serbia; 5 Department of Primary Health Care and Public Health, Faculty of Medicine, University of East Sarajevo, 73300 Foca, Bosnia and Herzegovina; 6 Institute for Medical Statistics and Informatics, Faculty of Medicine, University of Belgrade, 11000 Belgrade, Serbia

**Keywords:** postpartum hypertension, NSAID, acetaminophen

## Abstract

**Background/Objectives**: Nonsteroidal anti-inflammatory drugs (NSAIDs) are commonly used for postpartum pain management. However, previous studies have indicated that NSAIDs may increase systolic blood pressure, particularly in patients receiving antihypertensive therapy. The aim of the present study was to assess whether postpartum ibuprofen administration is associated with a higher risk of severe postpartum hypertension and increased mean arterial pressure (MAP) compared with acetaminophen. **Methods**: A systematic literature search was conducted in PubMed, Scopus, and Web of Science to identify relevant studies. Only randomized controlled trials were considered eligible for inclusion. For dichotomous outcomes, effect sizes were expressed as risk ratios (RRs) with corresponding 95% confidence intervals (CIs). For continuous outcomes, mean differences (MDs) with 95% CIs were calculated. Statistical heterogeneity among studies was assessed using the I^2^ statistic. A fixed-effects model was applied in cases of low heterogeneity (I^2^ < 20%). **Results**: No significant difference was observed in the prevalence of severe postpartum hypertension between the ibuprofen and acetaminophen groups (RR 1.07, 95% CI 0.84 to 1.35; *p* = 0.59; I^2^ = 0%). Similarly, MAP did not differ significantly between groups (MD −0.05 mmHg, 95% CI −1.53 to 1.42; *p* = 0.94; I^2^ = 0%). **Conclusions**: No increased risk of postpartum hypertension or difference in mean arterial pressure was observed between the ibuprofen and acetaminophen groups, supporting the safety of ibuprofen for postpartum analgesia in women with hypertensive disorders of pregnancy.

## 1. Introduction

Nonsteroidal anti-inflammatory drugs (NSAIDs) are among the most commonly prescribed medications for postpartum pain management because of their analgesic and anti-inflammatory effects. Effective postpartum analgesia is particularly important, as inadequate pain control may impair maternal recovery, mobility, breastfeeding, and overall well-being. Evidence from a systematic review suggests that NSAIDs provide more effective pain relief than paracetamol within four hours after administration [1]. As a result, NSAIDs, including ibuprofen, are widely used in routine postpartum care.

Despite their analgesic benefits, concerns have been raised regarding the cardiovascular effects of NSAIDs, particularly their potential to increase blood pressure. Previous studies have indicated that NSAIDs may increase systolic blood pressure (SBP), particularly in patients receiving antihypertensive therapy [2,3]. Although the average increase in SBP is generally modest, a subset of patients may experience clinically relevant elevations, with higher doses and certain NSAID agents associated with greater increases [2,3]. These findings have raised concerns regarding NSAID use in women with hypertensive disorders of pregnancy (HDP), a population already at increased risk of severe postpartum hypertension and associated maternal complications.

Initial concerns regarding NSAID safety in this setting originated from an early randomized controlled trial (RCT), which reported significantly higher postpartum blood pressure in patients receiving ibuprofen compared with those receiving acetaminophen [4]. Consequently, the American College of Obstetricians and Gynecologists (ACOG) recommended avoiding NSAIDs for postpartum analgesia in women with preeclampsia or other hypertensive disorders of pregnancy [5]. However, subsequent evidence has been inconsistent. Several observational studies [6,7,8,9] and RCTs [4,10,11,12] have evaluated the relationship between postpartum NSAID use and blood pressure outcomes, with several studies reporting no significant increase in severe hypertension or adverse maternal outcomes associated with ibuprofen administration.

In addition, three systematic reviews have attempted to synthesize the available evidence [13,14,15]. Nevertheless, consensus remains limited because of important methodological limitations, including the inclusion of heterogeneous patient populations combining mild and severe HDP, differences in antihypertensive treatment protocols, variation in analgesic regimens, and a limited number of high-quality randomized trials. As a result, uncertainty persists regarding the safety of NSAID administration in patients at the highest risk, particularly those with severe antenatal hypertension or severe preeclampsia.

Recently, a new RCT evaluated the effect of postpartum ibuprofen on blood pressure outcomes in patients with HDP and severe antenatal hypertension [16]. This study contributes important new evidence to a clinically relevant and controversial topic. In light of these emerging data, the aim of the present systematic review and meta-analysis was to assess whether postpartum ibuprofen administration is associated with a higher risk of severe postpartum hypertension and increased mean arterial pressure (MAP) compared with acetaminophen.

## 2. Materials and Methods

This systematic review and meta-analyses were performed according to the Preferred Reporting Items for Systematic Reviews and Meta-Analyses (PRISMA) guidelines (Table S1) [17]. The study protocol was registered in the International Prospective Register of Systematic Reviews (PROSPERO) under the following number: CRD420261340648.

### 2.1. Eligibility Criteria

This systematic review included studies based on predefined eligibility criteria structured according to the PICO framework.

#### 2.1.1. Population

Studies including adult women (≥18 years) with hypertensive disorders of pregnancy (HDP), including gestational hypertension, preeclampsia, or chronic hypertension with superimposed preeclampsia, were eligible. Studies involving pregnant women without HDP or participants younger than 18 years were excluded.

#### 2.1.2. Intervention

Eligible studies evaluated postpartum administration of ibuprofen as an analgesic intervention.

#### 2.1.3. Comparators

The comparator group consisted of women receiving acetaminophen (paracetamol). Studies were included regardless of dosage, route of administration, or duration of treatment, provided that both ibuprofen and acetaminophen were administered during the postpartum period.

#### 2.1.4. Exclusion Criteria

Studies were excluded if ibuprofen was not administered postpartum, if acetaminophen was not used as a comparator, or if other analgesic regimens were used without a clear comparison between ibuprofen and acetaminophen. Studies in which the independent effects of ibuprofen or acetaminophen could not be isolated were also excluded.

#### 2.1.5. Study Design

Only randomized controlled trials (RCTs) were included. Case reports, case series, review articles, editorials, letters, animal studies, and in vitro studies were excluded. 

#### 2.1.6. Context

Studies conducted in any clinical setting (e.g., hospitals, maternity wards, outpatient care) were eligible. No restrictions were applied regarding geographic location, healthcare system, or mode of delivery (vaginal or cesarean section).

### 2.2. Information Sources and Search Strategy

A systematic literature search was conducted across multiple electronic databases (PubMed, Scopus, and Web of Science) from inception to 7 March 2026. The search strategy combined terms related to hypertensive disorders of pregnancy, postpartum period, ibuprofen, and acetaminophen. Both Medical Subject Headings (MeSH) and free-text terms were used. Additionally, reference lists of relevant studies and reviews were screened to identify any potentially eligible studies not captured by the database search. The PubMed search strategy is presented in Table S2. The full search strategy was published in the PROSPERO record.

### 2.3. Selection and Data Collection Process

Two independent reviewers screened titles and abstracts for eligibility using the Rayyan software [18] (https://www.rayyan.ai/, last accessed 17 May 2026), followed by full-text assessment of potentially relevant studies. Discrepancies were resolved through discussion or consultation with a third reviewer. Data extraction was performed independently by two reviewers using a standardized data extraction form. Extracted data included study characteristics, population details, intervention and comparator characteristics, and outcomes of interest, such as prevalence of postpartum hypertension, MAP values, diuresis, duration of postpartum hospital stay, and use of postpartum antihypertensive medications.

### 2.4. Effect Measures

For dichotomous outcomes, effect sizes were expressed as risk ratios (RRs) with corresponding 95% confidence intervals (CIs). For continuous outcomes, mean differences (MDs) with 95% CIs were calculated. The primary outcomes were risk of severe postpartum hypertension, defined as systolic blood pressure ≥ 160 mmHg or diastolic blood pressure ≥ 105 mmHg, and MAP values. MAP for the Triebwasser et al. study [12] was calculated using a standard formula incorporating systolic and diastolic blood pressure (SBP and DBP), as detailed in the Supplementary Files (Supplementary Equation (S1)).

### 2.5. Synthesis Methods and Statistical Analysis

A quantitative synthesis (meta-analysis) was performed when at least two studies reported comparable outcomes. Studies were assessed for eligibility for each synthesis based on the availability of outcome-specific data and the comparability of intervention and control groups. Extracted data were tabulated, and studies were included in each meta-analysis only if they reported sufficient data for the outcome of interest. Studies contributing data to multiple outcomes were included in all relevant analyses. Statistical heterogeneity was assessed using the I^2^ statistic and Cochran’s Q test. Pooled estimates were calculated using a fixed-effects model if heterogeneity was low (I^2^ < 20%). Results are presented using forest plots. Statistical significance was defined as a two-sided *p*-value < 0.05. Statistical analyses were performed using Review Manager (RevMan) Web (version 10.2.0; The Cochrane Collaboration).

### 2.6. Sensitivity Analysis

A leave-one-out sensitivity analysis was performed by sequentially removing each study and recalculating the pooled effect estimate to evaluate the robustness of the results.

### 2.7. Study Risk of Bias and Certainty Assessment

The quality of included randomized controlled trials was assessed at the outcome level using the Cochrane RoB-2 tool. The overall certainty of evidence was assessed following the GRADE framework.

## 3. Results

### 3.1. Study Selection

**Figure 1:**
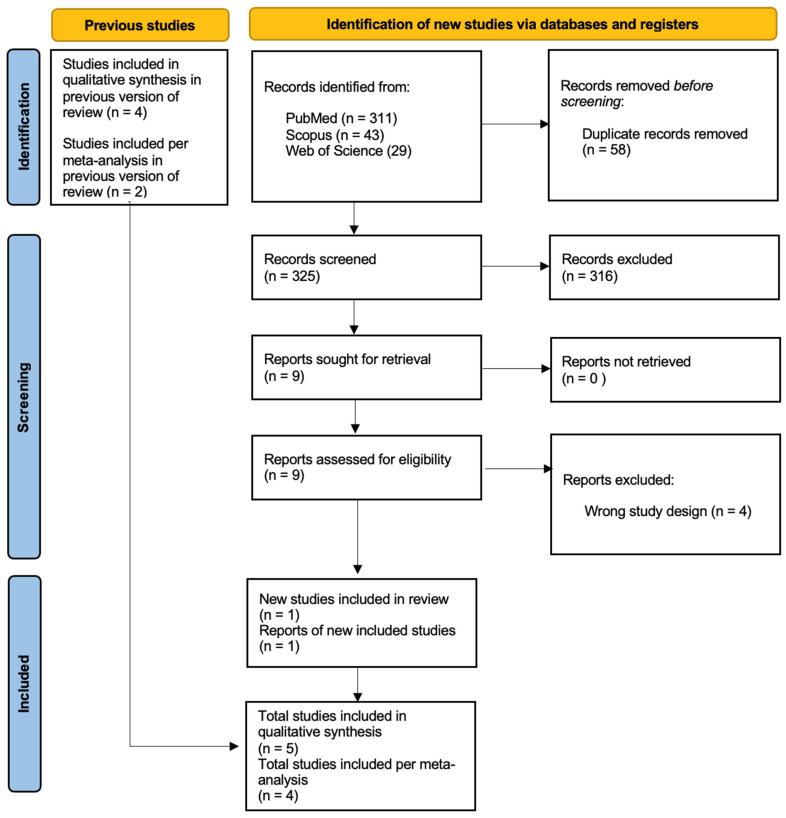
PRISMA flowchart.

The study selection process is summarized in the PRISMA flow diagram (Figure 1). A total of 383 records were identified through database searching. After the removal of duplicates, 325 records were screened based on title and abstract, of which nine underwent full-text review.

A total of five randomized controlled trials met the eligibility criteria and were included in the qualitative synthesis, of which four studies were included in the quantitative synthesis (meta-analysis).

### 3.2. Study Characteristics

The included studies comprised a total of 485 women with HDP, of whom 243 were allocated to the ibuprofen group and 242 to the acetaminophen group.

Four studies were conducted in the United States, and one study was conducted in Panama. All included studies were RCTs comparing postpartum administration of ibuprofen with acetaminophen for analgesia. Study characteristics are presented in Table S3.

### 3.3. Synthesis of Results

A quantitative synthesis (meta-analysis) was performed when at least two studies reported comparable outcomes. Statistical heterogeneity was assessed using the I^2^ statistic and Cochran’s Q test. Pooled estimates were calculated using a fixed-effects model if heterogeneity was low (I^2^ < 20%). Results are presented using forest plots. Statistical significance was defined as a two-sided *p*-value < 0.05. Statistical analyses were performed using Review Manager (RevMan) Web (version 10.2.0; The Cochrane Collaboration).

#### 3.3.1. Prevalence of Severe Postpartum Hypertension

**Figure 2:**
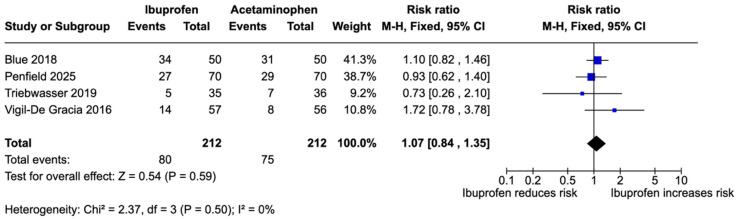
Risk of developing severe postpartum hypertension between the ibuprofen and acetaminophen groups [4,11,12,16]. Blue 2018 [11]; Penfield 2025 [16]; Triebwasser 2019 [12]; Vigil-De Gracia 2016 [4].

Four studies were included in the meta-analysis assessing the risk of severe postpartum hypertension. There was no significant difference between the ibuprofen and acetaminophen groups (RR 1.07, 95% CI 0.84 to 1.35; *p* = 0.59), with no observed heterogeneity (I^2^ = 0%) (Figure 2).

#### 3.3.2. Mean Arterial Pressure (MAP)

**Figure 3:**
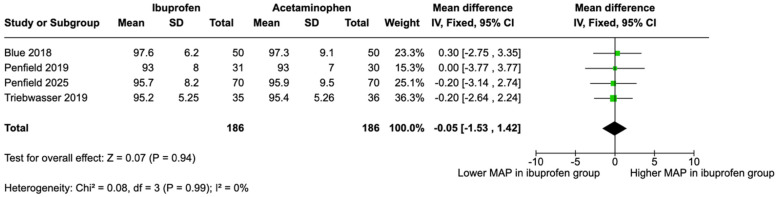
Differences in MAP between the ibuprofen and acetaminophen groups [10,11,12,16]. Blue 2018 [11]; Penfield 2019 [10]; Penfield 2025 [16]; Triebwasser 2019 [12].

Four studies were included in the meta-analysis evaluating MAP. There was no significant difference between groups (MD −0.05 mmHg, 95% CI −1.53 to 1.42; *p* = 0.94), with no evidence of heterogeneity (I^2^ = 0%) (Figure 3).

### 3.4. Subgroup Analysis

**Figure 4:**
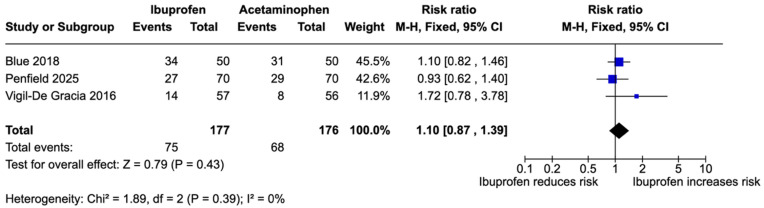
Subgroup analysis of women with severe HDP showing the risk of developing severe postpartum hypertension between the ibuprofen and acetaminophen groups [4,11,16]. Blue 2018 [11]; Penfield 2025 [16]; Vigil-De Gracia 2016 [4].

A subgroup analysis including only studies enrolling women with severe HDP was performed. For risk of severe postpartum hypertension, the pooled estimate remained non-significant (RR 1.10, 95% CI 0.87 to 1.39; *p* = 0.43), with no heterogeneity (I^2^ = 0%) (Figure 4).

**Figure 5:**
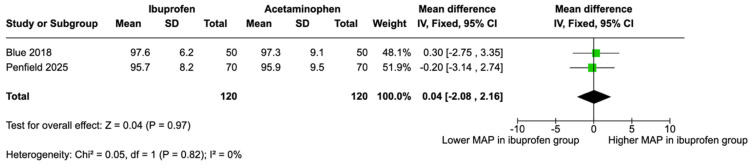
Subgroup analysis of women with severe HDP showing differences in MAP between the ibuprofen and acetaminophen groups [11,16]. Blue 2018 [11]; Penfield 2025 [16].

Similarly, for MAP, no significant difference was observed (MD 0.04 mmHg, 95% CI −2.08 to 2.16; *p* = 0.97), with no heterogeneity (I^2^ = 0%) (Figure 5).

### 3.5. Sensitivity Analysis

Leave-one-out sensitivity analysis demonstrated that sequential exclusion of individual studies did not alter the pooled effect estimates for any outcome, indicating that the results were robust (Figures S1 and S2). 

### 3.6. Risk of Bias

The risk of bias assessment using the Cochrane Risk of Bias tool (ROB 2) is presented in Figure S3. Risk of bias was assessed across five domains: randomization process (D1), deviations from intended interventions (D2), missing outcome data (D3), outcome measurement (D4), and selective reporting of results (D5). Overall, the included studies were judged to have a low risk of bias across most domains. However, one study demonstrated a high risk of bias. The study by Vigil de Gracia et al. [4] was assessed as having some concerns in domain D2 and a high risk of bias in domain D4. While this introduces a degree of uncertainty at the study level, the overall robustness of the findings is supported by the consistency of results across the included studies.

### 3.7. Certainty of Evidence

The certainty of the evidence for the effect of ibuprofen versus acetaminophen on severe postpartum hypertension was rated as moderate. Among the included studies, only one study was judged to have a high risk of bias, while the remaining studies were low risk, so no overall downgrade was applied for study limitations. The results were consistent across studies, and the population, intervention, and outcomes directly addressed our PICO question, so no downgrade was applied for inconsistency or indirectness. Imprecision was considered serious because the confidence interval for the pooled risk ratio (RR 1.07, 95% CI 0.84 to 1.35) was wide and crossed the threshold of no effect, and the total sample size was relatively small, leading to a one-level downgrade. This reflects the limited precision of the estimate due to the relatively small total sample size and the uncertainty around both potential benefit and harm within the confidence interval. Publication bias was not formally assessed due to the small number of included studies, and because the trials were prospectively registered and relatively large, no downgrade was applied for publication bias. Overall, these considerations led to a final GRADE rating of moderate certainty (Table S4).

## 4. Discussion

In this meta-analysis of RCTs including postpartum women with HDP, we found that ibuprofen use did not significantly increase the risk of postpartum hypertension and that there were no significant differences in MAP between the two groups. These findings indicate that ibuprofen is generally safe for postpartum analgesia in this population, with no evidence of clinically meaningful increases in blood pressure or need for additional antihypertensive therapy.

The hypertensive effects of NSAIDs are thought to result from several interrelated pathophysiological mechanisms involving renal, vascular, and neurohormonal regulation of blood pressure. NSAIDs inhibit cyclooxygenase (COX) enzymes, thereby reducing the synthesis of prostaglandins, particularly prostacyclin (PGI_2_) and prostaglandin E_2_ (PGE_2_), which normally promote vasodilation and maintain renal blood flow [19,20]. In the kidneys, prostaglandins play an important role in preserving glomerular filtration and facilitating sodium excretion, especially under conditions characterized by increased activation of the renin–angiotensin–aldosterone system or impaired renal perfusion [20,21]. Suppression of these prostaglandins by NSAIDs may therefore lead to sodium and water retention, increased intravascular volume, and subsequent elevations in blood pressure [19,20,21]. In addition, reduced prostaglandin-mediated vasodilation may increase systemic vascular resistance and enhance the vasoconstrictive effects of circulating catecholamines and angiotensin II [19,22]. NSAID administration has also been associated with attenuation of the antihypertensive effects of several commonly used medications, including beta-blockers, angiotensin-converting enzyme inhibitors, and diuretics, further contributing to blood pressure elevation [2,3]. These mechanisms may be particularly relevant in women with hypertensive disorders of pregnancy, who already exhibit endothelial dysfunction, increased vascular reactivity, and altered fluid balance. Consequently, NSAID-induced changes in renal hemodynamics and vascular tone may theoretically exacerbate postpartum hypertension and increase the risk of severe hypertensive complications in this vulnerable population.

When compared with previous studies, our results are consistent with three earlier RCTs evaluating NSAID use in postpartum women with HDP, which similarly found no significant effect on blood pressure or antihypertensive requirements [10,11,12]. The only RCT showing a modest difference was the trial by Vigil-De Gracia; however, the difference was evident only when systolic blood pressure was above 150 mmHg, and no effect was observed when BP exceeded 160 mmHg [4]. This nuance suggests that any observed differences are unlikely to be clinically significant and do not contradict the overall safety of ibuprofen in the postpartum setting.

Observational studies and prospective cohorts provide complementary evidence. Wasden et al. reported no difference in MAP or antihypertensive use among women exposed to NSAIDs postpartum [8]. Premkumar et al. found no association between the amount of NSAID use and outpatient blood pressure control [23]. Longitudinal data from Mukhtarova et al. and Ditisheim et al. highlight that postpartum blood pressure peaks around days 3–7 and a substantial proportion of women remain hypertensive at 6 weeks [24,25], underscoring that the absence of harm from ibuprofen occurs even in the context of natural postpartum BP fluctuations. Previous studies often reported antihypertensive therapy rather than the actual prevalence of postpartum severe hypertension, making our meta-analysis the first to provide more robust prevalence-based evidence from RCTs in this population.

These findings have important clinical implications. Women with HDP remain at high risk for long-term cardiovascular disease, including chronic hypertension, coronary artery disease, stroke, and heart failure [26,27]. Ensuring safe postpartum pain management is therefore essential. NSAIDs, particularly ibuprofen, are commonly used as part of multimodal analgesic regimens to reduce opioid exposure [28], and our results provide reassurance that their use does not worsen postpartum hypertension in this high-risk population. Multimodal strategies incorporating NSAIDs have also been shown to reduce opioid consumption without compromising pain control or increasing hospital stay [24]. In addition, evidence-based guidance emphasizes the importance of careful postpartum BP monitoring, individualized treatment decisions, and the need for robust thresholds to minimize maternal morbidity and mortality [29]. Collectively, these data support the inclusion of ibuprofen in postpartum analgesic protocols without increasing the risk of elevated blood pressure or antihypertensive medication requirements [8,23].

These findings align with prior RCT evidence, provide more robust prevalence-based data than previous observational studies, and support the continued use of NSAIDs as a safe and effective component of postpartum pain management. Future research should examine longer-term cardiovascular outcomes, optimal dosing strategies, and applicability across diverse healthcare settings to further inform postpartum analgesic guidelines [25]. Similarly, clinical markers such as the onset of diuresis can safely guide the duration of postpartum therapy in women with severe preeclampsia, highlighting the potential for individualized monitoring strategies in high-risk populations [30].

Strengths of this meta-analysis include the exclusive inclusion of RCTs, which minimizes bias compared with observational studies; the addition of a recently published trial, increasing sample size and statistical power; and rigorous methodology with standardized outcome definitions and comprehensive literature synthesis. These factors enhance the certainty of evidence compared with previous reviews, which often combined heterogeneous populations or relied on surrogate outcomes. Limitations include the relatively small number of trials and participants, which may have reduced statistical power and limited the precision of pooled effect estimates. Although no significant differences were observed between ibuprofen and acetaminophen, the confidence intervals crossed the line of no effect, indicating that modest differences cannot be entirely excluded. Therefore, the absence of statistical significance should not be interpreted as definitive equivalence between interventions. Nevertheless, the observed effect estimate for MAP was minimal and unlikely to represent a clinically meaningful difference. Furthermore, there is a predominance of studies from high-income countries, which may limit generalizability to lower-resource settings. Future large-scale RCTs are warranted to further clarify whether small differences in postpartum hypertension risk may exist.

## 5. Conclusions

No increased risk of postpartum hypertension or difference in MAP was observed between the ibuprofen and acetaminophen groups, supporting no evidence of increased risk associated with ibuprofen use for postpartum analgesia in women with HDP.

## Data Availability

The original contributions presented in this study are included in the article. Further inquiries can be directed to the corresponding author.

## Supplementary Materials

The following supporting information can be downloaded at: https://www.mdpi.com/article/10.3390/jcm15114042/s1, Table S1: PRISMA checklist, Table S2: PubMed search strategy, Table S3: Study characteristics, Table S4: Grade assessment, Supplementary Equation (S1), Figure S1: Sensitivity analysis of the risk of severe postpartum hypertension comparing ibuprofen versus acetaminophen, Figure S2: Sensitivity analysis of the difference in MAP between ibuprofen and acetaminophen group, Figure S3: Risk of bias assessment.
