# Supplementary material for: Ibuprofen vs. Acetaminophen After Delivery in Women with Hypertensive Disorders of Pregnancy: An Updated Systematic Review and Meta-Analysis of Randomized Controlled Trials
Source: J Clin Med. 2026 May 23;15(11):4042. doi: 10.3390/jcm15114042 (PMC13257922; doi:10.3390/jcm15114042)
Supplement: Supplementary file 1 [file jcm-15-04042-s001.zip › jcm-4296579-supplementary.pdf]

Table S1. PRISMA checklist

Section and Topic	Item #	Checklist item	Location where item is reported
**TITLE**			
Title	1	Identify the report as a systematic review.	p. 1
**ABSTRACT**			
Abstract	2	See the PRISMA 2020 for Abstracts checklist.	p. 1
**INTRODUCTION**			
Rationale	3	Describe the rationale for the review in the context of existing knowledge.	p. 2
Objectives	4	Provide an explicit statement of the objective(s) or question(s) the review addresses.	p. 2 (lines 63-67)
**METHODS**			
Eligibility criteria	5	Specify the inclusion and exclusion criteria for the review and how studies were grouped for the syntheses.	p. 2, 3 (lines 73-95)
Information sources	6	Specify all databases, registers, websites, organisations, reference lists and other sources searched or consulted to identify studies. Specify the date when each source was last searched or consulted.	p. 3 (lines 103-111)
Search strategy	7	Present the full search strategies for all databases, registers and websites, including any filters and limits used.	Table S2
Selection process	8	Specify the methods used to decide whether a study met the inclusion criteria of the review, including how many reviewers screened each record and each report retrieved, whether they worked independently, and if applicable, details of automation tools used in the process.	p. 3 lines 112-120
Data collection process	9	Specify the methods used to collect data from reports, including how many reviewers collected data from each report, whether they worked independently, any processes for obtaining or confirming data from study investigators, and if applicable, details of automation tools used in the process.	p. 3 lines 112-120
Data items	10a	List and define all outcomes for which data were sought. Specify whether all results that were compatible with each outcome domain in each study were sought (e.g. for all measures, time points, analyses), and if not, the methods used to decide which results to collect.	p. 3 lines (112-128)
	10b	List and define all other variables for which data were sought (e.g. participant and intervention characteristics, funding sources). Describe any assumptions made about any missing or unclear information.	p. 3 lines (112-128)
Study risk of bias assessment	11	Specify the methods used to assess risk of bias in the included studies, including details of the tool(s) used, how many reviewers assessed each study and whether they worked independently, and if applicable, details of automation tools used in the process.	p. 4 (lines 144-147)
Effect measures	12	Specify for each outcome the effect measure(s) (e.g. risk ratio, mean difference) used in the synthesis or presentation of results.	p. 3 (lines 121-128)
Synthesis methods	13a	Describe the processes used to decide which studies were eligible for each synthesis (e.g. tabulating the study intervention characteristics and comparing against the planned groups for each synthesis (item #5)).	p. 3 (lines 129-140)
	13b	Describe any methods required to prepare the data for presentation or synthesis, such as handling of missing summary statistics, or data	p. 3 (lines 129-

Section and Topic	Item #	Checklist item	Location where item is reported
		conversions.	140), Supplementary Equation 1
	13c	Describe any methods used to tabulate or visually display results of individual studies and syntheses.	p. 3 (lines 129-140)
	13d	Describe any methods used to synthesize results and provide a rationale for the choice(s). If meta-analysis was performed, describe the model(s), method(s) to identify the presence and extent of statistical heterogeneity, and software package(s) used.	p. 3 (lines 129-140)
	13e	Describe any methods used to explore possible causes of heterogeneity among study results (e.g. subgroup analysis, meta-regression).	p. 3 (lines 129-140)
	13f	Describe any sensitivity analyses conducted to assess robustness of the synthesized results.	p. 4 (Lines 141-143)
Reporting bias assessment	14	Describe any methods used to assess risk of bias due to missing results in a synthesis (arising from reporting biases).	NA
Certainty assessment	15	Describe any methods used to assess certainty (or confidence) in the body of evidence for an outcome.	p. 4 (lines 144-147)
**RESULTS**			
Study selection	16a	Describe the results of the search and selection process, from the number of records identified in the search to the number of studies included in the review, ideally using a flow diagram.	Figure 1, p. 4 (lines 149-156)
	16b	Cite studies that might appear to meet the inclusion criteria, but which were excluded, and explain why they were excluded.	Figure 1, p. 4 (lines 149-156)
Study characteristics	17	Cite each included study and present its characteristics.	Table S3, p. 4 (lines 157-162)
Risk of bias in studies	18	Present assessments of risk of bias for each included study.	Figure S3, p. 7 (lines 204-210)
Results of individual studies	19	For all outcomes, present, for each study: (a) summary statistics for each group (where appropriate) and (b) an effect estimate and its precision (e.g. confidence/credible interval), ideally using structured tables or plots.	Figures 2-5, Table S2
Results of syntheses	20a	For each synthesis, briefly summarise the characteristics and risk of bias among contributing studies.	p. 5-7 (lines 165-194)
	20b	Present results of all statistical syntheses conducted. If meta-analysis was done, present for each the summary estimate and its precision (e.g. confidence/credible interval) and measures of statistical heterogeneity. If comparing groups, describe the direction of the effect.	p. 5-7 (lines 165-194)
	20c	Present results of all investigations of possible causes of heterogeneity among study results.	p. 5-7 (lines 165-194)
	20d	Present results of all sensitivity analyses conducted to assess the robustness of the synthesized results.	Figure S2,

Section and Topic	Item #	Checklist item	Location where item is reported
			Figure S3, p. 6 (lines 195-198)
Reporting biases	21	Present assessments of risk of bias due to missing results (arising from reporting biases) for each synthesis assessed.	*NA
Certainty of evidence	22	Present assessments of certainty (or confidence) in the body of evidence for each outcome assessed.	Table S4, p. 7 (lines 211-224)
**DISCUSSION**			
Discussion	23a	Provide a general interpretation of the results in the context of other evidence.	p. 8 (lines 225-271)
	23b	Discuss any limitations of the evidence included in the review.	p. 8-9 (271-279)
	23c	Discuss any limitations of the review processes used.	p. 8-9
	23d	Discuss implications of the results for practice, policy, and future research.	p. 8 (lines 264-271)
**OTHER INFORMATION**			
Registration and protocol	24a	Provide registration information for the review, including register name and registration number, or state that the review was not registered.	p. 2 (lines 68-72)
	24b	Indicate where the review protocol can be accessed, or state that a protocol was not prepared.	p. 2 (lines 68-72)
	24c	Describe and explain any amendments to information provided at registration or in the protocol.	*NA
Support	25	Describe sources of financial or non-financial support for the review, and the role of the funders or sponsors in the review.	p. 9 (lines 296-298)
Competing interests	26	Declare any competing interests of review authors.	p. 9 (line 303)
Availability of data, code and other materials	27	Report which of the following are publicly available and where they can be found: template data collection forms; data extracted from included studies; data used for all analyses; analytic code; any other materials used in the review.	p. 9 (lines 301-302)

From: Page MJ, McKenzie JE, Bossuyt PM, Boutron I, Hoffmann TC, Mulrow CD, et al. The PRISMA 2020 statement: an updated guideline for reporting systematic reviews. BMJ 2021;372:n71. doi: 10.1136/bmj.n71. This work is licensed under CC BY 4.0. To view a copy of this license, visit https://creativecommons.org/licenses/by/4.0/

*NA: Not applicable

Table S2. PubMed search strategy

Concept	Keywords / MeSH Terms	Boolean Operators / Notes
Hypertension in pregnancy	"hypertension"[MeSH Terms], "hypertension"[All Fields], ("hypertensive"[All Fields] AND "disorder"[All Fields]), "hypertensive disorder"[All Fields]	OR
Pregnancy	"pregnancy"[MeSH Terms], "pregnancy"[All Fields], "pregnancies"[All Fields], "pregnancy s"[All Fields]	OR
Pregnancy-induced hypertension / Gestational hypertension	"hypertension, pregnancy induced"[MeSH Terms], ("hypertension"[All Fields] AND "pregnancy induced"[All Fields]), "pregnancy-induced hypertension"[All Fields], ("gestational"[All Fields] AND "hypertension"[All Fields]), "gestational hypertension"[All Fields]	OR
Preeclampsia	"pre eclampsia"[MeSH Terms], "pre eclampsia"[All Fields], "preeclampsia"[All Fields]	OR
NSAIDs / Anti-inflammatory drugs	"anti inflammatory agents non steroidal"[Pharmacological Action], "anti inflammatory agents non steroidal"[Supplementary Concept], "anti inflammatory agents non steroidal"[All Fields], "nsaid"[All Fields], "anti inflammatory agents, non steroidal"[MeSH Terms], ("anti inflammatory"[All Fields] AND "agents"[All Fields] AND "non steroidal"[All Fields]), "non steroidal anti inflammatory agents"[All Fields], "nsaids"[All Fields], "nsaid s"[All Fields], ("ibuprofen"[Supplementary Concept] OR "ibuprofen"[All Fields] OR "ibuprofen"[MeSH Terms] OR "ibuprofen s"[All Fields] OR "ibuprofens"[All Fields])	OR
Postpartum period	"postpartum period"[MeSH Terms], ("postpartum"[All Fields] AND "period"[All Fields]), "postpartum period"[All Fields], "postpartum"[All Fields]	OR
Postpartum hypertension / blood pressure	"hypertense"[All Fields], "hypertension"[MeSH Terms], "hypertension"[All Fields], "hypertension s"[All Fields], "hypertensions"[All Fields], "hypertensive"[All Fields], "hypertensive s"[All Fields], "hypertensives"[All Fields], "blood pressure"[MeSH Terms], ("blood"[All Fields] AND "pressure"[All Fields]), "blood pressure"[All Fields], "blood pressure determination"[MeSH Terms], ("blood"[All Fields] AND "pressure"[All Fields] AND "determination"[All Fields]), "blood pressure determination"[All Fields], "arterial pressure"[MeSH Terms], ("arterial"[All Fields] AND "pressure"[All Fields]), "arterial pressure"[All Fields]	OR

Table S3. Study characteristics

Author and Year	Country	Hypertensive Disorder	Intervention	Control	Number of Participants	Prevalence of Postpartum Hypertension, n (%)	Use of Postpartum Antihypertensive Medications	Average Postpartum MAP, mean (SD), mmHg	Diuresis	Length of Postpartum Hospital Stay
Penfield 2025 [16]	USA	Severe HDP	600 mg ibuprofen every 6 h	Acetaminophen 650 mg every 6 h	I: 70, C: 70	I: 27 (38.6); C: 29 (41.4)	Any: I: 25 (35.7%); C: 28 (40%); Severe episode: I: 14 (20%), C: 21 (30%)	I: 95.7 (8.2); C: 95.9 (9.5)	I: 45 (64.7%); C: 46 (65.7%)	I: 2.3 ± 0.7; C: 2.3 ± 0.7
Penfield 2019 [10]	USA	Mild/Moderate HDP	600 mg ibuprofen every 6 h	Acetaminophen 650 mg every 6 h	I: 31, C: 30	NA	NA	I: 93 (8); C: 93 (7)	I: 61%; C: 77%	I: 48h; C: 43h
Blue 2018 [11]	USA	Preeclampsia with severe features	600 mg ibuprofen every 6 h	Acetaminophen 650 mg every 6 h	I: 50, C: 50	Any postpartum BP ≥160/110 mm Hg: I: 34 (68); C: 31 (62)	Any postpartum meds for acute BP control: I: 30 (60); C: 26 (52)	I: 97.6 (6.2); C: 97.3 (9.1)	NA	I: 3.8 (1.4); C: 4.0 (1.3)
Triebwasser 2019 [12]	USA	Gestational hypertension or preeclampsia without severe features	600 mg ibuprofen every 6 h	Acetaminophen 650 mg every 6 h	I: 35, C: 36	I: 5 (14.3); C: 7 (19.4)	NA	I: 95.2 (5.25); C: 95.4 (5.26)	NA	NA
Vigil-De Gracia 2016 [4]	Panama	Severe pre-eclampsia	400 mg ibuprofen every 8 h	Acetaminophen 1 g every 6 h	I: 57, C: 56	>150/100 mmHg: I: 28.6% (16/56); C: 63.1% (36/57); Severe hypertension (>160/110 mmHg between 24–96 h postpartum) not significantly different: 14.5% vs 24.5%	NA	NA	NA	NA

Supplementary Equation (S1).

$$\mathrm{MAP}_{mean} = \frac{\mathrm{SBP}_{mean} + 2 \cdot \mathrm{DBP}_{mean}}{3}$$

$$\mathrm{MAP}_{SD} = \sqrt{\frac{SD_{SBP}^2 + 4 \cdot SD_{DBP}^2}{9}}$$

Figure S1. Sensitivity analysis of the risk of severe postpartum hypertension comparing ibuprofen versus acetaminophen [4,11,12,16].

A)

	Ibuprofen		Acetaminophen			Risk ratio	Risk ratio
**Study or Subgroup**	**Events**	**Total**	**Events**	**Total**	**Weight**	**M-H, Fixed, 95% CI**	**M-H, Fixed, 95% CI**
✖ Blue 2018	34	50	31	50	0.0%	1.10 [0.82 , 1.46]	
✔ Penfield 2025	27	70	29	70	66.0%	0.93 [0.62 , 1.40]	
✔ Triebwasser 2019	5	35	7	36	15.7%	0.73 [0.26 , 2.10]	
✔ Vigil-De Gracia 2016	14	57	8	56	18.4%	1.72 [0.78 , 3.78]	
**Total**		**162**		**162**	**100.0%**	**1.04 [0.74 , 1.47]**	
Total events:	46		44				

Test for overall effect: Z = 0.25 (P = 0.80)

Heterogeneity: Chi² = 2.28, df = 2 (P = 0.32); I² = 12%

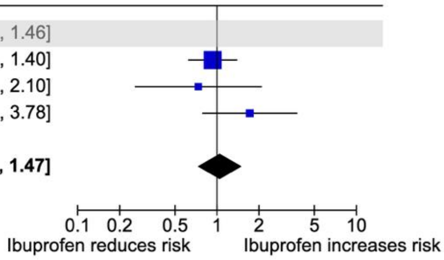

B)

	Ibuprofen		Acetaminophen			Risk ratio	Risk ratio
**Study or Subgroup**	**Events**	**Total**	**Events**	**Total**	**Weight**	**M-H, Fixed, 95% CI**	**M-H, Fixed, 95% CI**
✔ Blue 2018	34	50	31	50	67.4%	1.10 [0.82 , 1.46]	
✖ Penfield 2025	27	70	29	70	0.0%	0.93 [0.62 , 1.40]	
✔ Triebwasser 2019	5	35	7	36	15.0%	0.73 [0.26 , 2.10]	
✔ Vigil-De Gracia 2016	14	57	8	56	17.6%	1.72 [0.78 , 3.78]	
**Total**		**142**		**142**	**100.0%**	**1.15 [0.87 , 1.53]**	
Total events:	53		46				

Test for overall effect: Z = 0.98 (P = 0.33)

Heterogeneity: Chi² = 1.81, df = 2 (P = 0.40); I² = 0%

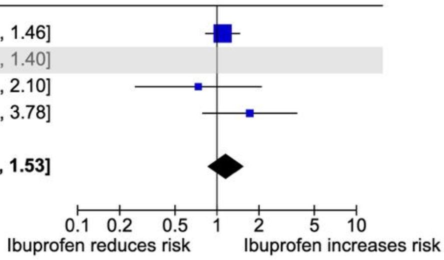

C)

	Ibuprofen		Acetaminophen			Risk ratio	Risk ratio
**Study or Subgroup**	**Events**	**Total**	**Events**	**Total**	**Weight**	**M-H, Fixed, 95% CI**	**M-H, Fixed, 95% CI**
✔ Blue 2018	34	50	31	50	45.5%	1.10 [0.82 , 1.46]	
✔ Penfield 2025	27	70	29	70	42.6%	0.93 [0.62 , 1.40]	
✖ Triebwasser 2019	5	35	7	36	0.0%	0.73 [0.26 , 2.10]	
✔ Vigil-De Gracia 2016	14	57	8	56	11.9%	1.72 [0.78 , 3.78]	
**Total**		**177**		**176**	**100.0%**	**1.10 [0.87 , 1.39]**	
Total events:	75		68				

Test for overall effect: Z = 0.79 (P = 0.43)

Heterogeneity: Chi² = 1.89, df = 2 (P = 0.39); I² = 0%

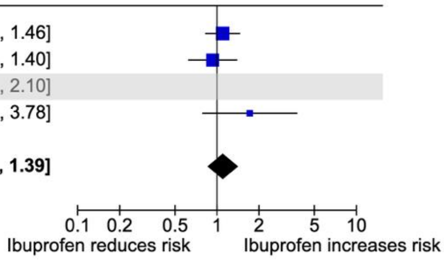

D)

	Ibuprofen		Acetaminophen			Risk ratio	Risk ratio
**Study or Subgroup**	**Events**	**Total**	**Events**	**Total**	**Weight**	**M-H, Fixed, 95% CI**	**M-H, Fixed, 95% CI**
✔ Blue 2018	34	50	31	50	46.3%	1.10 [0.82 , 1.46]	
✔ Penfield 2025	27	70	29	70	43.3%	0.93 [0.62 , 1.40]	
✔ Triebwasser 2019	5	35	7	36	10.3%	0.73 [0.26 , 2.10]	
✖ Vigil-De Gracia 2016	14	57	8	56	0.0%	1.72 [0.78 , 3.78]	
**Total**		**155**		**156**	**100.0%**	**0.99 [0.78 , 1.26]**	
Total events:	66		67				

Test for overall effect: Z = 0.10 (P = 0.92)

Heterogeneity: Chi² = 0.89, df = 2 (P = 0.64); I² = 0%

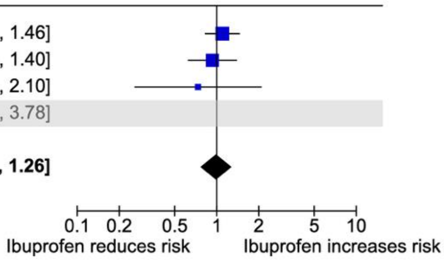

Figure S2. Sensitivity analysis of the difference in MAP between ibuprofen and acetaminophen group [10-12,16].

A)

Study or Subgroup	Ibuprofen Mean	Ibuprofen SD	Ibuprofen Total	Acetaminophen Mean	Acetaminophen SD	Acetaminophen Total	Weight	Mean difference IV, Fixed, 95% CI
✖ Blue 2018	97.6	6.2	50	97.3	9.1	50	0.0%	0.30 [-2.75 , 3.35]
✔ Penfield 2019	93	8	31	93	7	30	19.9%	0.00 [-3.77 , 3.77]
✔ Penfield 2025	95.7	8.2	70	95.9	9.5	70	32.7%	-0.20 [-3.14 , 2.74]
✔ Triebwasser 2019	95.2	5.25	35	95.4	5.26	36	47.3%	-0.20 [-2.64 , 2.24]
**Total**			**136**			**136**	**100.0%**	**-0.16 [-1.84 , 1.52]**

Test for overall effect: Z = 0.19 (P = 0.85)

Heterogeneity: Chi² = 0.01, df = 2 (P = 1.00); I² = 0%

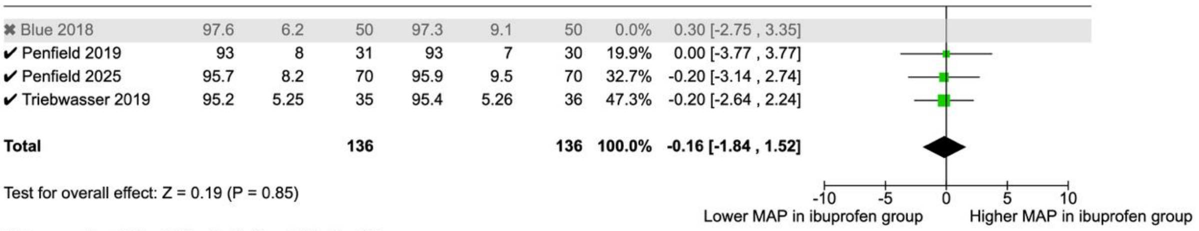

B)

Study or Subgroup	Ibuprofen Mean	Ibuprofen SD	Ibuprofen Total	Acetaminophen Mean	Acetaminophen SD	Acetaminophen Total	Weight	Mean difference IV, Fixed, 95% CI
✔ Blue 2018	97.6	6.2	50	97.3	9.1	50	27.5%	0.30 [-2.75 , 3.35]
✖ Penfield 2019	93	8	31	93	7	30	0.0%	0.00 [-3.77 , 3.77]
✔ Penfield 2025	95.7	8.2	70	95.9	9.5	70	29.6%	-0.20 [-3.14 , 2.74]
✔ Triebwasser 2019	95.2	5.25	35	95.4	5.26	36	42.9%	-0.20 [-2.64 , 2.24]
**Total**			**155**			**156**	**100.0%**	**-0.06 [-1.66 , 1.54]**

Test for overall effect: Z = 0.08 (P = 0.94)

Heterogeneity: Chi² = 0.07, df = 2 (P = 0.96); I² = 0%

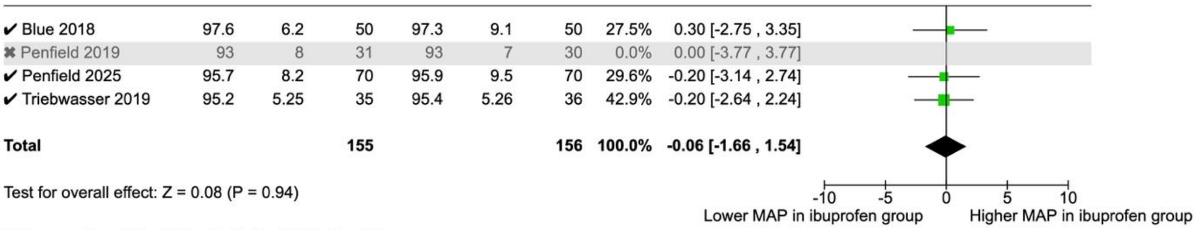

C)

Study or Subgroup	Ibuprofen Mean	Ibuprofen SD	Ibuprofen Total	Acetaminophen Mean	Acetaminophen SD	Acetaminophen Total	Weight	Mean difference IV, Fixed, 95% CI
✔ Blue 2018	97.6	6.2	50	97.3	9.1	50	31.1%	0.30 [-2.75 , 3.35]
✔ Penfield 2019	93	8	31	93	7	30	20.4%	0.00 [-3.77 , 3.77]
✖ Penfield 2025	95.7	8.2	70	95.9	9.5	70	0.0%	-0.20 [-3.14 , 2.74]
✔ Triebwasser 2019	95.2	5.25	35	95.4	5.26	36	48.5%	-0.20 [-2.64 , 2.24]
**Total**			**116**			**116**	**100.0%**	**-0.00 [-1.71 , 1.70]**

Test for overall effect: Z = 0.00 (P = 1.00)

Heterogeneity: Chi² = 0.06, df = 2 (P = 0.97); I² = 0%

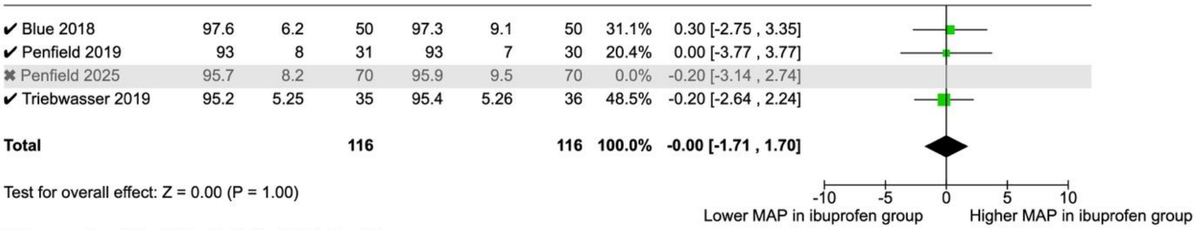

D)

Study or Subgroup	Ibuprofen Mean	Ibuprofen SD	Ibuprofen Total	Acetaminophen Mean	Acetaminophen SD	Acetaminophen Total	Weight	Mean difference IV, Fixed, 95% CI
✔ Blue 2018	97.6	6.2	50	97.3	9.1	50	36.6%	0.30 [-2.75 , 3.35]
✔ Penfield 2019	93	8	31	93	7	30	24.0%	0.00 [-3.77 , 3.77]
✔ Penfield 2025	95.7	8.2	70	95.9	9.5	70	39.4%	-0.20 [-3.14 , 2.74]
✖ Triebwasser 2019	95.2	5.25	35	95.4	5.26	36	0.0%	-0.20 [-2.64 , 2.24]
**Total**			**151**			**150**	**100.0%**	**0.03 [-1.82 , 1.88]**

Test for overall effect: Z = 0.03 (P = 0.97)

Heterogeneity: Chi² = 0.05, df = 2 (P = 0.97); I² = 0%

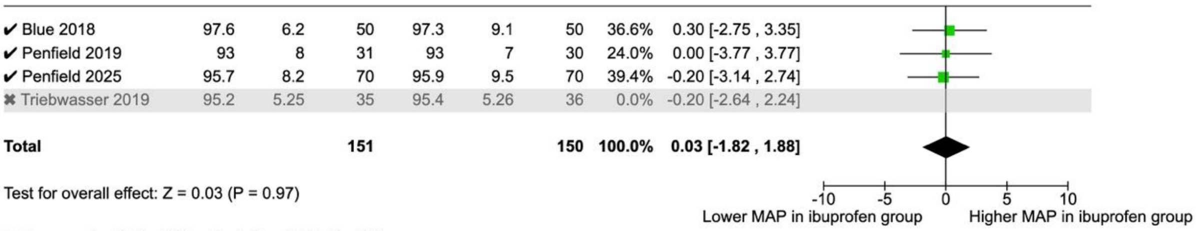

Table S4. GRADE assessment.

Outcome	Effect	Participants	Risk of Bias[1]	Inconsistency[2]	Indirectness[3]	Imprecision[4]	Publication Bias[5]	Certainty
Postpartum hypertension	RR: 1.07 [0.84, 1.35]	I: 212<br>C: 212	Low	I^2=0% (No concerns)	No concerns	Serious	Not assessed	⊕⊕⊕◯ Moderate
MAP	MD:-0.05 [-1.53, 1.42]	I: 186,<br>C: 186	Low	I^2=0% (No concerns)	No concerns	Serious	Not assessed	⊕⊕⊕◯ Moderate

[1] Risk of bias: Assessed per study using Cochrane ROB 2.0; summarized per outcome.

[2] Inconsistency: Based on I^2 and forest plot inspection. Downgraded if $I^2 > 50\%$ or conflicting point estimates.

[3] Indirectness: All studies included women with HDP, used relevant interventions, comparators, and outcomes

[4] Imprecision: Downgraded if CI crosses no effect or sample size is small. Ranges from Not serious, Serious to Very serious.

[5] Publication bias: Not formally assessed because fewer than 10 studies per comparison.

Figure S3. Risk of bias assessment [4, 10-12, 16].

Study ID	Experimental	Comparator	Outcome	Weight	D1	D2	D3	D4	D5	Overall
Penfield 2019	Ibuprofen	Acetaminophen	postpartum hypertension, MAP	1	+	+	+	+	+	+
Blue 2018	Ibuprofen	Acetaminophen	postpartum hypertension, MAP	1	+	+	+	+	+	+
Triebwasser 2019	Ibuprofen	Acetaminophen	postpartum hypertension, MAP	1	+	+	+	+	+	+
Penfield 2025	Ibuprofen	Acetaminophn	postpartum hypertension, MAP	1	+	+	+	+	+	+
Vigil-De Gracia 2016	Ibuprofen	Acetaminophen	postpartum hypertension, MAP	1	+	!	+	-	+	-

+ Low risk
! Some concerns
- High risk

D1 Randomisation process
D2 Deviations from the intended interventions
D3 Missing outcome data
D4 Measurement of the outcome
D5 Selection of the reported result
